# A Poly(methacrolein-*co*-methacrylamide)-Based Template Anchoring Strategy for the Synthesis of Fluorescent Molecularly Imprinted Polymer Nanoparticles for Highly Selective Serotonin Sensing

**DOI:** 10.3390/nano15130977

**Published:** 2025-06-24

**Authors:** Madhav Biyani, Mizuki Matsumoto, Yasuo Yoshimi

**Affiliations:** 1 Chemical Engineering Laboratory, Shibaura Institute of Technology, 3-7-5 Toyosu, Koto-Ku, Tokyo 135-8548, Japan; am22102@sic.shibaura-it.ac.jp (M.B.);; 2 Innovative Global Program, Shibaura Institute of Technology, 3-7-5 Toyosu, Koto-Ku, Tokyo 135-8548, Japan

**Keywords:** solid-phase synthesis, fluorescent molecularly imprinted polymer nanoparticles (fMIP-NPs), polymer-anchored immobilization, serotonin

## Abstract

Neurotransmitters such as serotonin regulate key physiological and cognitive functions, yet real-time detection remains challenging due to the limitations of conventional techniques like amperometry and microdialysis. Fluorescent molecularly imprinted polymer nanoparticles (fMIP-NPs) offer a promising alternative and are typically synthesized via solid-phase synthesis, in which template molecules are covalently immobilized on a solid support to enable site-specific imprinting. However, strong template–template interactions during this process can compromise selectivity. To overcome this, we incorporated a poly(methacrolein-*co*-methacrylamide)-based template anchoring strategy to minimize undesired template interactions and enhance imprinting efficiency. We optimized the synthesis of poly(methacrolein-*co*-methacrylamide) under three different conditions by varying the monomer compositions and reaction parameters. The poly(methacrolein-*co*-methacrylamide) synthesized under Condition 3 (5:1 methacrolein-to-methacrylamide molar ratio, 1:150 initiator-to-total monomer ratio, and 4.59 M total monomer concentration) yielded the most selective fMIP-NPs, whose fluorescence intensity increased with an increase in serotonin concentration, rising by up to 37% upon serotonin binding. This improvement is attributed to higher aldehyde functionality in the poly(methacrolein-*co*-methacrylamide) which enhances template immobilization and generates a rigid imprinted cavity to interact with serotonin. These findings suggest that the developed fMIP-NPs hold significant potential as imaging probes for neurotransmitter detection, contributing to advanced studies in neural network analysis.

## 1. Introduction

In the animal brain, neurotransmitters play an important role in coordinating a diverse array of physiological and cognitive functions [1,2]. These specialized chemicals serve as the primary means of communication between neurons, facilitating the transmission of signals across synaptic junctions [3]. By modulating the strength and frequency of neuronal signaling, neurotransmitters govern processes ranging from basic motor functions to complex cognitive tasks, including emotion regulation, learning, and memory formation [4,5]. The monitoring of neural signals facilitates the mapping of neural circuits, elucidating the complex networks of interconnected neurons responsible for specific functions and behaviors [6,7]. Neurotransmitters are commonly detected using either amperometric [8,9,10,11,12] or microdialysis methods [13,14,15,16,17,18,19,20]. The amperometric method involves the insertion of a microfiber electrode into the brain to detect the redox current of neurotransmitters, while the microdialysis method utilizes a hollow fiber membrane to collect neurotransmitters and pump them into a detector. However, each method has its limitations: the amperometric method lacks selectivity, while the microdialysis method cannot provide real-time data. To overcome these limitations, both analysis methods are often employed concurrently, with the data integrated to infer neurotransmitter behavior. Despite advances in detection techniques, determining the exact location and timing of neurotransmitter secretion remains challenging. Thus, there is a need for imaging probes that can be used to visualize neurotransmitter release in real-time. Continuous efforts have been made to develop imaging techniques for visualizing neurotransmitter secretion using protein probes. For example, Wang et al. discussed the development of genetically encoded fluorescent sensors, which represent a significant advancement in the field of neuroimaging [21]. These sensors are engineered proteins that fluoresce when they bind to specific neurotransmitters or neuromodulators, enabling the real-time, in vivo monitoring of neurotransmitter dynamics within living organisms. While these genetically encoded sensors offer high specificity and are less invasive compared to traditional electrode-based methods, they require a long period of time (several months) to obtain enough data for one experiment. Several researchers have attempted to create protein probes using genome editing techniques, but these efforts have been met with limited success [22,23,24,25,26,27,28,29]. A molecularly imprinted polymer (MIP) is a polymer designed with specific recognition sites or cavities that can selectively bind to templates with high affinity and specificity, and can be prepared in a shorter period of time than protein probes [30]. Solid-phase synthesis is a method for preparing molecularly imprinted polymer nanoparticles (MIP-NPs), in which the target molecule is covalently immobilized on a glass bead surface as a template, as established by Piletsky and his colleagues [31,32]. We have applied the solid-phase synthesis to prepare MIP-NPs containing fluorescent functional groups (fluorescent MIP-NPs: fMIP-NPs) as imaging probes, with fluorescence intensity depending on the concentration of the template. In a previous study, the template was immobilized on the glass bead surface by anchoring it through aminoalkylsilane [33]. From that study, we clarified that interactions among template molecules reduce the affinity between the template and the functional monomer during polymerization, thereby decreasing the selectivity of the synthesized MIP. To minimize interactions among the template molecules, our approach involves the polymer-anchored immobilization of the template using poly(methacrolein-*co*-methacrylamide) as the polymer anchor. The use of soft anchors, such as hydrophilic polymers, for template immobilization provides both structural support and flexibility to the template molecules, thereby minimizing undesired interactions among them. In this work, we synthesized poly(methacrolein-*co*-methacrylamide) under three different conditions, varying the comonomer ratios and reaction parameters. Serotonin was immobilized on glass beads via the poly(methacrolein-*co*-methacrylamide) anchor, which then served as a template for the synthesis of fMIP-NPs. The fMIP-NPs were subsequently evaluated for their fluorescence sensitivity to the target.

## 2. Materials and Methods

**Figure 1:**
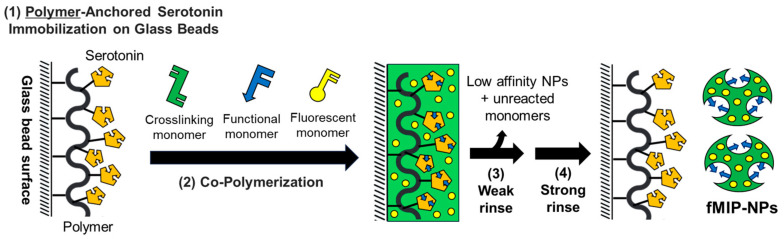
Scheme of the procedure for the synthesis of fMIP-NPs with the template anchored by a polymer on glass beads: (1) The target molecule (serotonin) was immobilized on the surface of glass beads through a polymer anchor, serving as the template for the molecularly imprinted polymer (MIP). (2) A functional monomer, a crosslinking monomer, and a fluorescent monomer were copolymerized in the vicinity of the surface. (3) The resulting copolymer, which has a low affinity for the template, along with any unreacted monomers, was removed using a weaker rinse. (4) The fluorescent molecularly imprinted polymer nanoparticles (fMIP-NPs), which have a high affinity for the template, were then dissociated from the surface using a stronger rinse.

In this work, the fMIP-NPs were prepared using the procedure illustrated in Figure 1. The general process for preparation involves four main steps: (1) the immobilization of the template anchored by the polymer on the glass bead surface; (2) the copolymerization of the functional, crosslinking, and fluorescent monomers; (3) weak rinsing to remove low-affinity nanoparticles and unreacted monomers; and (4) strong rinsing to dissociate the fMIP-NPs from the glass bead surface.

### 2.1. Chemicals

Serotonin (5-hydroxytryptamine: 5-HT) hydrochloride, methacrylic acid (MAA), ethylene glycol dimethacrylate (EDMA), 0.1 mol/L of potassium permanganate solution, and iron(III) chloride hexahydrate were purchased from Fujifilm Wako Pure Chemical Corporation (Osaka, Japan). L-tryptophan (L-Trp) was purchased from Peptide Institute, Inc. (Osaka, Japan). Methacrolein (stabilized with hydroquinone), methacrylamide, benzyldiethyldithiocarbamate (BDDC), 2,2′-azobis[2-(2-imidazolin-2-yl)propane] dihydrochloride, sodium dodecyl sulfate (SDS), and 3-aminopropyltrimethoxysilane (3-APTMS) were purchased from Tokyo Chemical Industry Co., Ltd. (Tokyo, Japan). Glass beads with a diameter of 50 µm (Rolloblast^®^) were purchased from Renfert GmbH (Hilzingen, Germany). Diallyl fluorescein (DAF), a fluorescent monomer, was synthesized according to the procedure described by Liu et al. [34]. Sodium hydroxide, and N,N-dimethylformamide (DMF) were purchased from Kanto Chemical Co., Inc. (Tokyo, Japan). Deuterium oxide (D_2_O, 100.0 atom% D) was purchased from Thermo Scientific Chemicals (Tokyo, Japan).

### 2.2. Synthesis of Poly(methacrolein-co-methacrylamide) (Poly(MAL-co-MAM))

Poly(methacrolein-*co*-methacrylamide) was selected as a polymeric anchor for serotonin immobilization on the surface of glass beads due to its unique functional properties. The incorporation of methacrolein monomers provides reactive aldehyde groups, enabling covalent crosslinking with the primary amine of serotonin through Schiff base formation. This aldehyde functionality is critical for the stable and efficient immobilization of serotonin onto the glass bead surface. In addition, methacrylamide was incorporated into the copolymer to enhance its water solubility, a necessary characteristic for performing immobilization experiments in aqueous media.

To optimize the copolymer properties, poly(methacrolein-*co*-methacrylamide) was synthesized under three different conditions, varying the comonomer ratios and reaction parameters. The synthesized copolymers were characterized and evaluated for their efficacy in immobilizing serotonin on glass beads. The details of the synthesis conditions are shown in Table 1.

#### 2.2.1. Distillation of Methacrolein

To enhance the stabilization of methacrolein at a high temperature, an additional 0.4 g of hydroquinone was added to 40 mL of methacrolein solution stabilized with hydroquinone, serving as a polymerization inhibitor. A standard distillation apparatus was set up, and the solution was heated using an oil bath set to 90 °C while stirring with a magnetic stirrer. The boiling point of methacrolein is 69 °C, and the vapors formed were continuously monitored with a thermometer. When the vapor temperature reached 62–64 °C, condensation into liquid droplets was observed. The initial distillate, containing low-boiling impurities, was collected and discarded. Once the vapor temperature stabilized at 65–67 °C, the purified methacrolein distillate was collected separately in another receiving flask. The purified methacrolein solution, free from the polymerization inhibitor, was obtained from the receiving flask for further use.

#### 2.2.2. Radical Co-Polymerization of Methacrolein and Methacrylamide

**Figure 2:**
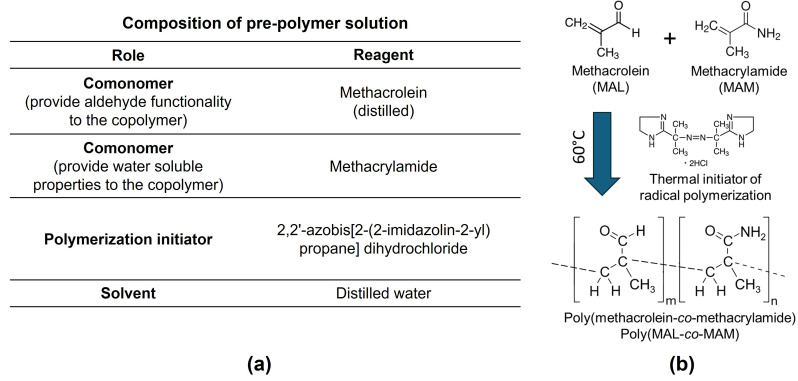
(**a**) Composition of the pre-polymer solution, including the comonomers, polymerization initiator, solvent, and respective roles. (**b**) Radical copolymerization of Methacrolein and Methacrylamide using a thermal initiator at 60 °C.

For the synthesis of poly(methacrolein-*co*-methacrylamide), methacrolein, methacrylamide and 2,2′-azobis[2-(2-imidazolin-2-yl)propane] dihydrochloride (thermal initiator of radical polymerization, [35]) were mixed according to the synthesis conditions shown in Table 1. The mixture was then diluted with 30 mL of distilled water, which remained consistent across all conditions. To create an oxygen-free environment, nitrogen gas was bubbled through distilled water before being passed into the reaction solution, facilitating the removal of dissolved oxygen and maintaining an inert atmosphere. The reaction mixture was then heated in an oil bath set to 60 °C to initiate the free-radical copolymerization. (See Figure 2). The copolymerization was carried out overnight to ensure the complete conversion of the comonomers. The resulting copolymer was subsequently collected by sedimentation through dropwise addition into vigorously stirred methanol and then washed with methanol using a glass suction filter. The synthesized copolymer was characterized by ¹H NMR spectroscopy using deuterium oxide (D_2_O) as the solvent, and by redox titration under acidic conditions using 1 mM of potassium permanganate (KMnO_4_) solution as the oxidizing agent.

### 2.3. Pretreatment of Glass Beads for the Template Immobilization

**Figure 3:**
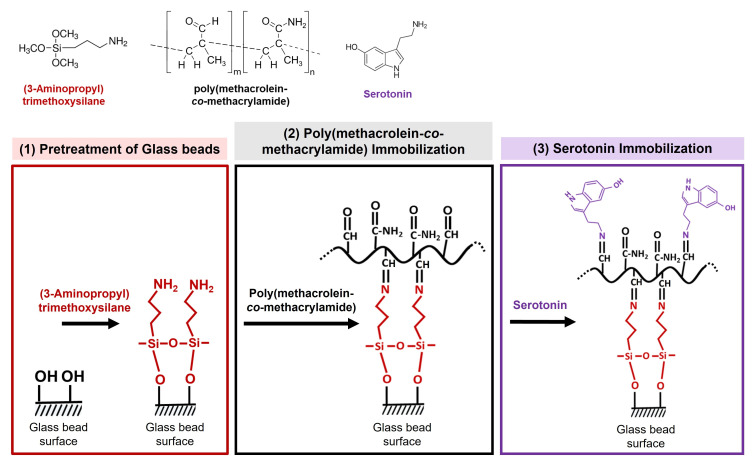
Scheme of the procedure for poly(methacrolein-*co*-methacrylamide)-anchored serotonin immobilization on the glass bead surface: (1) Pretreatment of glass beads—Primary amine groups are introduced onto the glass bead surface through silane treatment using 3-APTMS. (2) Poly(methacrolein-*co*-methacrylamide) immobilization—The copolymer is immobilized on the aminated glass beads via the reaction between its aldehyde groups and the primary amine groups on the glass bead surface. (3) Serotonin immobilization—Serotonin is immobilized on the glass bead surface through the reaction of its primary amine group with the aldehyde groups of the copolymer.

A total of 80 g of the 50 µm glass beads was dissolved in a 4 M aqueous solution of sodium hydroxide in a round bottom flask. The solution was boiled with continuous stirring for 2 h. After heating, the solution was filtered through a 20 µm sieve to collect the glass beads, which were then washed with distilled water until the pH of the supernatant was neutral. The glass beads were further washed with 2000 mL of distilled water, followed by 500 mL of methanol, using a glass suction filter, and dried under a vacuum overnight. The glass beads were collected through a 100 µm sieve and heat-dried at 180 °C in nitrogen atmosphere overnight. Next, the glass beads were soaked in a dehydrated toluene solution of (3-Aminopropyl)trimethoxysilane (3-APTMS) and heated at 90 °C with continuous stirring for 24 h to introduce a regulated density of amino groups on their surface.  (See Figure 3(1)) The solution was filtered through a 20 µm sieve to collect the glass beads, which were then washed with methanol to remove silane coupling aggregates. The glass beads were further washed with 500 mL of methanol using a glass suction filter and dried under a vacuum overnight.

### 2.4. Immobilization of Poly(Methacrolein-co-methacrylamide) on Pre-Treated Glass Beads

A total of 500 mg of synthesized poly(methacrolein-*co*-methacrylamide) was dissolved in 50 mL of distilled water. The aminated glass beads were added to the solution. The suspension of the beads was continuously stirred for 24 h to allow the aldehyde groups of the copolymer to react with the amino group on the beads. ((See Figure 3(2)) The glass beads were washed with 1000 mL of distilled water, followed by 200 mL of methanol, using a glass suction filter, and were dried under a vacuum overnight. After drying, the poly(methacrolein-*co*-methacrylamide)-immobilized glass beads were passed through a 100 µm sieve to ensure a uniform bead size.

### 2.5. Immobilization of Serotonin on Poly(Methacrolein-co-methacrylamide)-Immobilized Glass Beads

A total of 8.71 mg of 5-hydroxytryptamine hydrochloride (serotonin) was dissolved in 50 mL of distilled water to prepare a 0.819 mM serotonin solution. To this, 5 g of poly(methacrolein-*co*-methacrylamide)-immobilized glass beads were added. The solution was continuously stirred for 24 h to allow the aldehyde groups of the copolymer immobilized on the glass beads to react with the primary amino group of serotonin. (See Figure 3(3)). The copolymer serves as a cross-linker. The glass beads were washed with 200 mL of distilled water, followed by 100 mL of methanol, using a glass suction filter, and were dried under a vacuum overnight. After drying, the serotonin-immobilized glass beads were passed through a 100 µm sieve to ensure a uniform bead size. The immobilization of serotonin on glass beads was confirmed using 0.01 M of aqueous ferric chloride solution as an indicator.

### 2.6. Synthesis of fMIP-NPs for 5-HT (Serotonin)

A total of 3.0 g of serotonin-immobilized glass beads, methacrylic acid (MAA, 360 µL), ethylene glycol dimethacrylate (EDMA, 1.29 g, 6.49 mmol), benzyl diethyldithiocarbamate (BDDC, 0.15 g, 0.62 mmol), diallyl fluorescein (DAF, 10 mg, 0.24 mmol), distilled water (900 µL), and DMF (6 mL) were mixed in a quartz tube and ultrasonicated for 10 min. The suspension was pre-bubbled with nitrogen for 20 min to remove dissolved oxygen, followed by irradiation using a Xe lamp. The light source, coupled to an optical fiber, was positioned 1 cm below the quartz tube and fixed at its center, with nitrogen bubbling maintained throughout the 20-min irradiation process. The co-polymerized glass beads were packed into a 60 mL solid-phase extraction column with a polyethylene frit (Spelco SPE Tube, Sigma-Aldrich, St. Louis, MO, USA) for the multi-step release of fMIP-NPs from the beads. Initially, 100 mL of 25% DMF solution was passed through the packed beads using the pressure of air supplied from a syringe to release and collect low-affinity nanoparticles and impurities. Subsequently, 100 mL of 8 mM sodium dodecyl sulfate (SDS) solution was passed through the beads using the air pressure again to release and collect the fMIP-NPs. The medium of the eluted dispersions was exchanged with pH 7.4 phosphate-buffered saline (PBS) using a tubular cellulosic dialysis membrane (Sekisui Material Solutions, Tokyo, Japan: 17 mm in diameter, 5 nm in pore size). To prepare the dialysis membrane, it was immersed in deionized water and ultrasonicated for 10 min to remove glycerin. The collected fluids were poured into membrane tubes clipped at both ends and immersed in a stirred 2000 mL of pH 7.4 PBS as a dialysate fluid. The dialysate was replaced every three hours with fresh PBS for three exchanges. After the dialysis, the solution inside the membrane was collected in a sample bottle wrapped with aluminum foil and stored in the refrigerator.

### 2.7. Synthesis of Fluoroscent Non Imprinted Polymer Nano Particles (fNIP-NPs)

The fluorescent non-imprinted polymer nanoparticles (fNIP-NPs) were synthesized using a procedure identical to that used for the fluorescent molecularly imprinted polymer nanoparticles (fMIP-NPs) (see Section 2.6), with one key difference: serotonin-absent poly(methacrolein-*co*-methacrylamide)-immobilized glass beads were used instead of serotonin-immobilized glass beads to omit the template molecule.

### 2.8. Evaluation of the Sensitivity and Selectivity of the fMIP-NPs and fNIP-NPs

The prepared dispersion of fMIP-NPs (or fNIP-NPs) in PBS was mixed with either PBS or a PBS solution of serotonin (template) or L-tryptophan (analog) at a volume ratio of 7:3. Fluorescence intensity measurements were performed using an FP-750 fluorospectrophotometer (JASCO, Hachioji, Japan) with an excitation wavelength of 425 nm, and emission was recorded between 500 and 540 nm. The dependence of the fluorescence intensity of fMIP-NPs or fNIP-NPs on the concentrations of serotonin and its analog was evaluated. All measurements were conducted at room temperature.

### 2.9. Particle Size and Morphological Characterization of fMIP-NPs

For particle size analysis, the fMIP-NP dispersion in PBS was mixed with PBS at a volume ratio of 7:3. Particle size measurements were performed using dynamic light scattering (DLS) spectroscopy (DelsaMax Pro, Beckman Coulter, Brea, CA, USA). For morphology analysis, 10 µL of the fMIP-NP dispersion was spread onto carbon tape and dried overnight at room temperature. The sample was then coated with a thin film of platinum/palladium using a sputter coater (Smart Coater, DII-29010SCTR, JEOL Ltd., Tokyo, Japan). The morphology of the fMIP-NPs was observed using scanning electron microscopy (SEM; JSM-6010LV, JEOL Ltd., Tokyo, Japan).

## 3. Results and Discussion

### 3.1. Characterization of Poly(Methacrolein-co-methacrylamide)

#### 3.1.1. ^1^H-NMR Analysis

**Figure 4:**
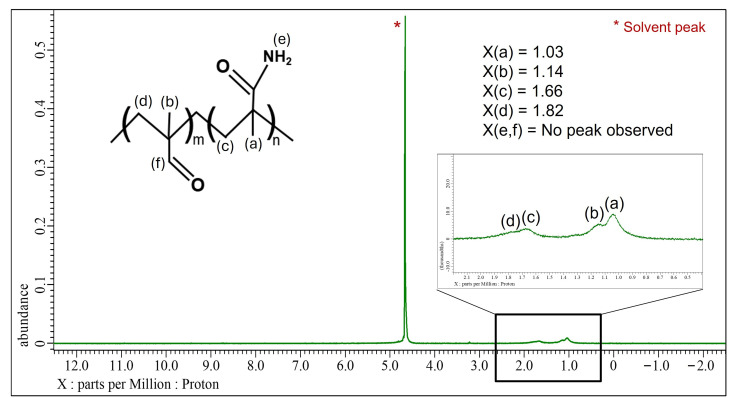
^1^H-NMR Spectrum of poly(methacrolein-*co*-methacrylamide) (Condition 1) in D_2_O. Proton signals are assigned as follows based on the chemical structure: (a) methyl protons from methacrylamide units at 1.03 ppm, (b) methyl protons from methacrolein units at 1.14 ppm, (c) methylene protons from methacrylamide units at 1.66 ppm, and (d) methylene protons from methacrolein units at 1.82 ppm. Peaks corresponding to (e) amide –NH_2_ and (f) aldehyde –CHO protons were not observed. The large peak at approximately 4.7 ppm (marked with *) corresponds to the residual solvent signal from D_2_O.

The ¹H-NMR spectrum of poly(methacrolein-*co*-methacrylamide) synthesized under Condition 1 is shown in Figure 4. The signals at 1.82 ppm (–CH_2_) and 1.14 ppm (–CH_3_) originate from the (–CH_2_C(CH_3_)–CHO) unit of the copolymer, while the signals at 1.66 ppm (–CH_2_) and 1.03 ppm (–CH_3_) originate from the (–CH_2_C(CH_3_)–NH_2_) unit of the copolymer. However, due to peak splitting, the –CH_2_ and –CH_3_ signals from both units overlapped, making them indistinguishable. The signal corresponding to –CHO was not observed, possibly due to the low concentrations of –CHO in the copolymer, which may be beyond the detection limit of the ¹H-NMR system [36]. Additionally, the signal corresponding to –NH_2_ was not observed due to rapid proton exchange with the D_2_O solvent [37,38,39]. To observe this peak, ¹H-NMR was attempted in a less exchange-prone solvent such as CDCl_3_ or DMSO-d_6_, but the copolymer was not soluble in either of these solvents. As a result, the copolymer’s characterization to determine the moles of aldehyde functionalities could not be achieved based on ¹H-NMR data. Therefore, redox titration using potassium permanganate (KMnO_4_) as an oxidizing agent was performed to quantify the aldehyde functionality in the copolymer.

#### 3.1.2. Quantification of Aldehyde Groups by KMnO_4_ Redox Titration

The aldehyde groups in the copolymer were quantified by redox titration, during which potassium permanganate (KMnO_4_ ) was reduced to Mn^2+^ in an acidic medium, while the aldehyde groups in the copolymer were oxidized to carboxylic acid groups, as shown in the following chemical equation: 2 MnO_4_^−^ + 5 -(CH_2_C(CH_3_)-CHO)- + 6 H^+^ → 2 Mn^2+^ + 5 -(CH_2_C(CH_3_)-COOH)- + 3 H_2_O. Based on the stoichiometry of the reaction, 2 moles of MnO_4_^−^ react with 5 moles of the aldehyde group, -(CH_2_C(CH_3_)-CHO)-. Therefore, the moles of -(CH_2_C(CH_3_)-CHO)- in the copolymer were calculated as 2.5 times the moles of KMnO_4_ that reacted, as determined from the volume of KMnO_4_ solution consumed at the titration end point. As shown in Table 2, poly(methacrolein-*co*-methacrylamide) synthesized under Condition 3, where the molar ratio of methacrolein to methacrylamide in the pre-polymer solution increased to 5:1 and the total comonomer concentration was raised to 4.59 M, resulted in the highest aldehyde functionality in the copolymer.

### 3.2. Confirmation of Serotonin Immobilization Using FeCl_3_ Solution as an Indicator

**Figure 5:**
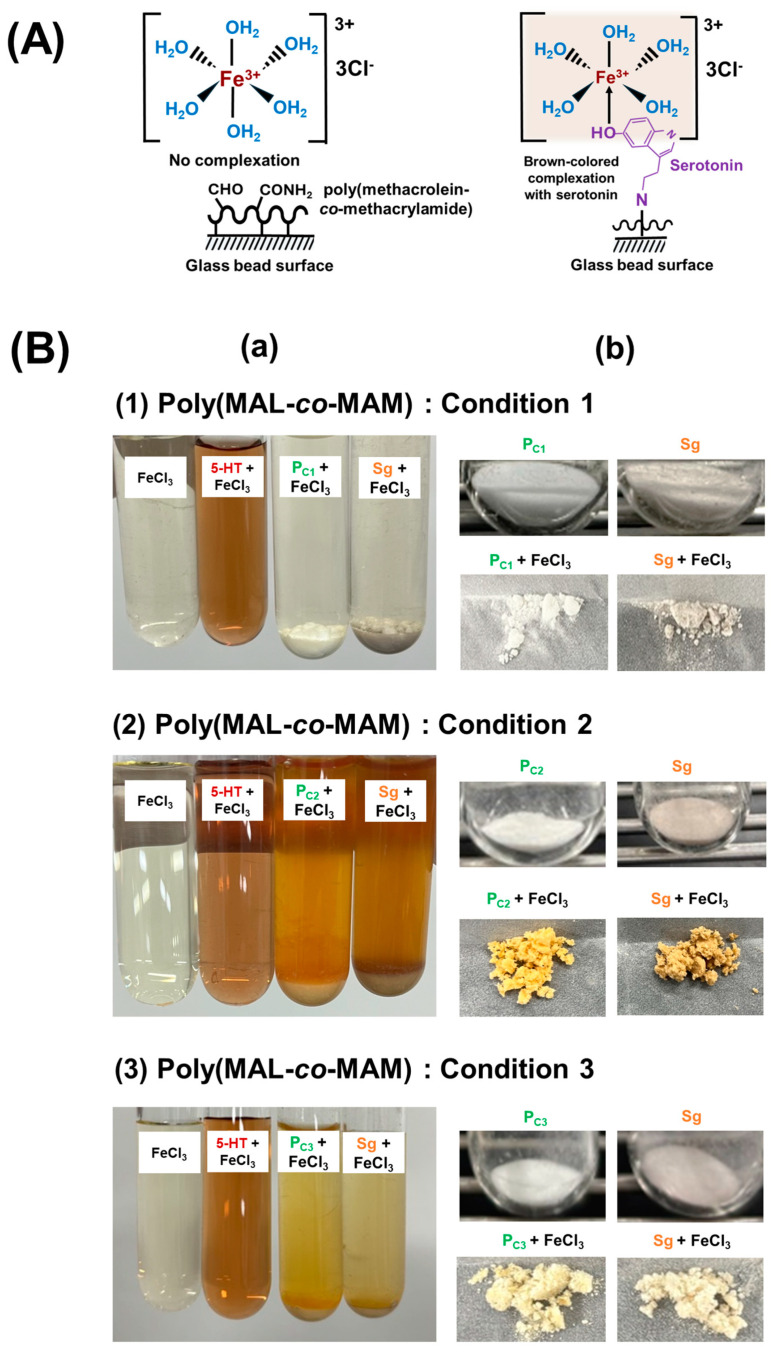
(**A**) Detection mechanism of serotonin immobilized on glass beads using FeCl_3_ solution. (**B**) (**a**) Solutions from left to right: 0.01 M FeCl_3_ solution, 0.01 g serotonin (5-HT) + FeCl_3_ solution, 0.5 g of poly(methacrolein-*co*-methacrylamide)-immobilized glass beads synthesized under Condition 1 (P_C1_; (**1**)) + FeCl_3_, Condition 2 (P_C2_; (**2**)) + FeCl_3_, Condition 3 (P_C3_; (**3**)) + FeCl_3_, and 0.5 g of serotonin-immobilized glass beads (Sg) + FeCl_3_. (**b**) Top row: poly(methacrolein-*co*-methacrylamide)-immobilized glass beads (P_C_) (left) and serotonin-immobilized glass beads (Sg) (right). Bottom row: filtered products of poly(methacrolein-*co*-methacrylamide)-immobilized glass beads + FeCl_3_ solution (left) and serotonin-immobilized glass beads + FeCl_3_ solution (right).

The successful immobilization of serotonin on glass beads was confirmed using an aqueous solution of FeCl_3_ (0.01 M) as an indicator. As shown in Figure 5A, on the left, when FeCl_3_ solution is introduced to poly(methacrolein-*co*-methacrylamide)-immobilized glass beads without serotonin, no interaction occurs between Fe^3+^ and the glass bead surface. As a result, Fe^3+^ remains in its hydrated complex form, [Fe(H_2_O)_6_]^3+^, leading to no characteristic coloration. However, on the right, when FeCl_3_ solution is introduced to serotonin-immobilized glass beads, the phenol group in the serotonin molecule acts as a ligand, interacting with Fe^3+^ ions in the presence of surrounding water molecules, resulting in a characteristic color change. The oxygen atom in the hydroxyl group of phenol has lone pairs of electrons that can participate in coordination with Fe^3+^, forming a ligand-to-metal charge transfer (LMCT) complex. This complexation process involves the transfer of electron density from the phenol oxygen to the Fe^3+^ center, resulting in a stable coordination structure. The LMCT interaction often induces a characteristic color change due to electronic transitions within the complex [40,41]. In the case of serotonin-immobilized glass beads, the phenol group of serotonin interacts with Fe^3+^ in the presence of water, forming a brown-colored LMCT complex, which serves as qualitative evidence of serotonin immobilization on glass beads.

Figure 5B shows the results of FeCl_3_ addition to glass beads under different synthesis conditions of poly(methacrolein-*co*-methacrylamide). For Condition 1 (refer to Figure 5B (1)), no significant color change was observed when FeCl_3_ was added to either the copolymer-immobilized glass beads or the serotonin-immobilized glass beads. The glass beads retained their original appearance before and after FeCl_3_ addition, indicating that serotonin immobilization could not be confirmed. This suggests that either serotonin was not immobilized or only a very low density of serotonin was immobilized, which is insufficient to produce the characteristic brown color. In contrast, for Condition 2 and Condition 3 (refer to Figure 5B (2) and (3), respectively), FeCl_3_ addition to copolymer-immobilized glass beads produced no coloration, but a characteristic brown color developed upon the addition to serotonin-immobilized glass beads. This brown coloration confirms successful serotonin immobilization under these conditions.

According to Table 2, for Condition 1, the aldehyde content in the copolymer was significantly lower, which likely resulted in a very low density of serotonin immobilized, which was insufficient to produce a detectable brown color with FeCl_3_. Conversely, for Condition 2 and Condition 3, the higher aldehyde content in the copolymer facilitated greater serotonin immobilization, enabling the formation of the characteristic brown complex upon FeCl_3_ addition.

However, it is important to note that while the aldehyde content was the highest for the copolymer synthesized under Condition 3, as confirmed by the titration experiment, the intensity of the brown coloration does not directly correlate with the amount of immobilized serotonin. This discrepancy may be attributed to the non-covalent adsorption of serotonin on the glass beads prepared in Condition 2, which is indicated by the significant color change in the supernatant fluid. A portion of the non-covalently adsorbed serotonin–Fe^3+^ complex may desorb from the surface of the glass beads due to its high solubility in water. The surface of the beads modified with the copolymer from Condition 2 is likely in a state that promotes serotonin adsorption primarily through non-covalent interactions. Therefore, the glass beads in Condition 3, which are richest in aldehyde groups, have lower total serotonin adsorption than those in Condition 2, but the proportion of non-covalent adsorption is smaller.

### 3.3. Sensitivity of fMIP-NPs of 5-HT

**Figure 6:**
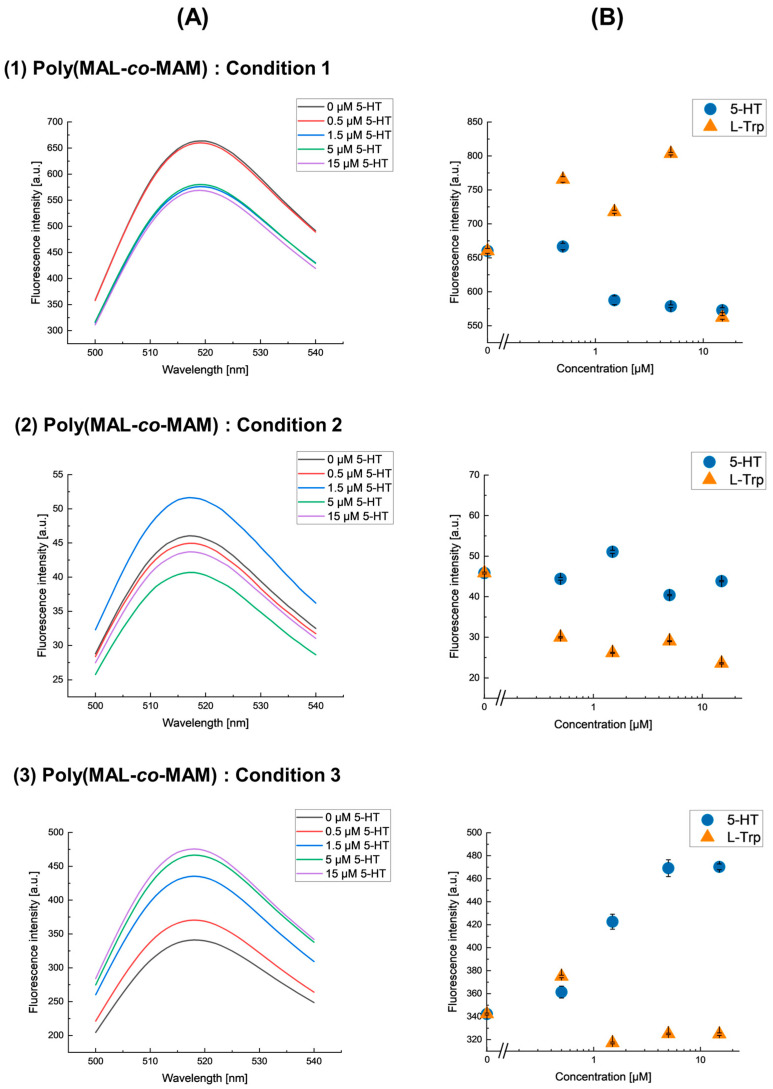
(**A**) Fluorescence emission spectra of fMIP-NPs synthesized using serotonin immobilized on glass beads via a poly(methacrolein-co-methacrylamide) anchor under three different conditions, (**1**) Condition 1, (**2**) Condition 2, and (**3**) Condition 3, each measured at varying concentrations of serotonin (5-HT). (**B**) Effect of serotonin (5-HT, circles) and its analog L-tryptophan (L-Trp, triangles) on the fluorescence intensity of the corresponding fMIP-NPs under each condition.

fMIP-NPs were synthesized using serotonin (5-HT) immobilized on the surface of glass beads via a poly(methacrolein-*co*-methacrylamide) anchor prepared under three different conditions. The fluorescence emission spectra of the fMIP-NPs at various serotonin concentrations under these conditions are shown in Figure 6A. The dependency of the fluorescence intensity on the concentrations of serotonin (5-HT) and its analog, L-tryptophan (L-Trp), is shown in Figure 6B.

The fluorescence intensity of fMIP-NPs synthesized using serotonin immobilized on glass beads via a poly(methacrolein-*co*-methacrylamide) anchor prepared under Condition 1 and Condition 2, which contain 0.77% and 1.59% aldehyde, respectively, showed no dependency of fluorescence intensity on either serotonin or L-tryptophan concentrations. In contrast, the fluorescence intensity of fMIP-NPs synthesized using serotonin immobilized on glass beads via a poly(methacrolein-*co*-methacrylamide) anchor prepared under Condition 3, which contains 7.14% aldehyde, was highly selective and sensitive to serotonin, with the fluorescence intensity of fMIP-NPs correlating positively with 5-HT concentration. The fluorescence intensity of the fMIP-NPs increased by a maximum of 37% in response to serotonin concentration. This enhanced sensitivity and selectivity are likely due to the higher number of aldehyde groups in the copolymer synthesized under Condition 3, which facilitates greater template immobilization on the glass bead surface while reducing non-specific interactions on the polymeric branches. Notably, the fluorescence intensity consistently peaked at 518 nm under all conditions, indicating that the excitation of the fluorescein moiety in fMIP-NP is not affected by interactions with serotonin. These results suggest that the copolymer synthesized under Condition 3 is optimal for producing highly selective fMIP-NPs. A comparative summary of the performance of these fMIP-NPs with previously reported MIPs for serotonin detection is provided in Table 3. The batch-to-batch reproducibility of the fluorescence response of fMIP-NPs was evaluated. In the initially optimized batch, a 37% increase in fluorescence response was observed, whereas a subsequent batch prepared after 8 months exhibited only a 7% increase (Supplementary Figure S1). Although reproducibility could be confirmed in terms of selectivity toward serotonin and a similar trend in fluorescence increase, a significant reduction in sensitivity was observed. This loss of sensitivity is likely attributable to the use of aged poly(methacrolein-*co*-methacrylamide) and serotonin during synthesis. The degradation of these materials over time may have reduced the number and quality of high-affinity binding sites formed during polymerization, thereby diminishing the sensitivity of the imprinted nanoparticles in this batch.

These findings suggest that fMIP-NPs hold significant potential as optically self-reporting probes for neurotransmitter detection and visualization, enabling the real-time monitoring of neurotransmitter release. While our current results provide preliminary validation, future research will focus on assessing the applicability of fMIP-NPs as imaging probes in living animal neurons, for example, in *Aplysia* microbrain. Such organisms serve as ideal models due to their relatively simple and well-characterized nervous systems, along with the presence of large, easily identifiable neurons that facilitate detailed studies of neuronal signaling and synaptic activity [42]. By combining fMIP-NPs with fluorescence microscopy and advanced imaging techniques, real-time monitoring of neurotransmitter dynamics and neural network activity could be achieved. However, further design for the efficient adsorption and targeted localization of fMIP-NPs onto neuronal surfaces is required. Poly(methacrolein-*co*-methacrylamide) is also a feasible tool for localization through crosslinking between the amino groups introduced into the fMIP-NPs and those present on neuronal membrane proteins. Amino groups can be readily introduced into fMIP-NPs by incorporating amino-functional monomers (e.g., allylamine) during the synthesis stage.

**Table 3:** Comparison of the performance of the developed fMIP-NPs with previously reported molecularly imprinted polymers (MIPs) for serotonin detection.

	Template Material	Imprinting	Detection Method	Sensitivity	Selectivity
This work	Serotonin immobilized via poly(methacrolein-*co*-methacrylamide) anchor	Surface	Fluorescence	37% ↑ (0–15 µM)	High selectivity (vs. L-Trp)
MIP 1 [33]	Serotonin immobilized via blended silane anchor	Surface	Fluorescence	5% ↑ (0–15 µM)	High selectivity (vs. L-Trp)
MIP 2 [43]	Serotonin	Bulk	Impedance	8.4% ↑ (0–64.5 nM)	High selectivity (vs. 5-HIAA and oxidized serotonin)

### 3.4. Insensitivity of fNIP-NPs

**Figure 7:**
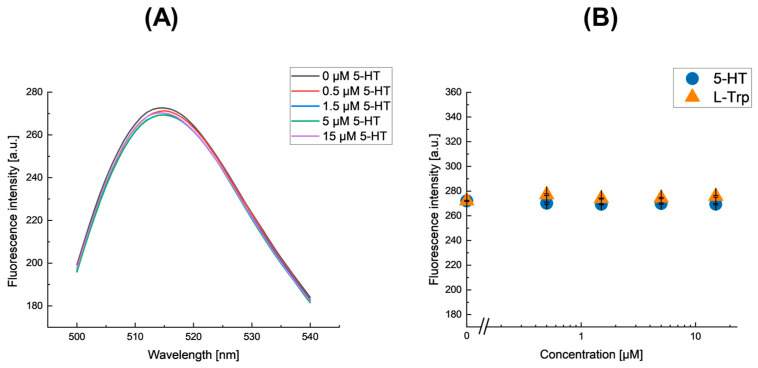
(**A**) Fluorescence emission spectra of fNIP-NPs synthesized using serotonin-absent poly(methacrolein-co-methacrylamide)-immobilized glass beads under Condition 3, each measured at varying concentrations of serotonin (5-HT). (**B**) Effect of serotonin (5-HT, circles) and its analog L-tryptophan (L-Trp, triangles) on the fluorescence intensity of the corresponding fNIP-NPs under this condition.

fNIP-NPs were synthesized as a control using serotonin-absent poly(methacrolein-*co*-methacrylamide)-immobilized glass beads prepared under Condition 3. The fluorescence emission spectrum of the fNIP-NPs at various serotonin concentrations is shown in Figure 7A. The dependence of fluorescence intensity on the concentrations of serotonin (5-HT) and its analog, L-tryptophan (L-Trp), is shown in Figure 7B.

The fluorescence intensity of fNIP-NPs was insensitive to both 5-HT and L-Trp concentrations. The imprinting factor, which is the ratio of the sensitivity of the MIP to that of the NIP, was too large to quantify. These results confirm that the fluorescence changes observed in fMIP-NPs are primarily due to specific interactions between serotonin-imprinted cavities and the analytes, highlighting the specificity of the molecular imprinting process. (See Table 3).

### 3.5. Characterization of fMIP-NPs

#### 3.5.1. Particle Size Analysis by Dynamic Light Scattering (DLS)

**Figure 8:**
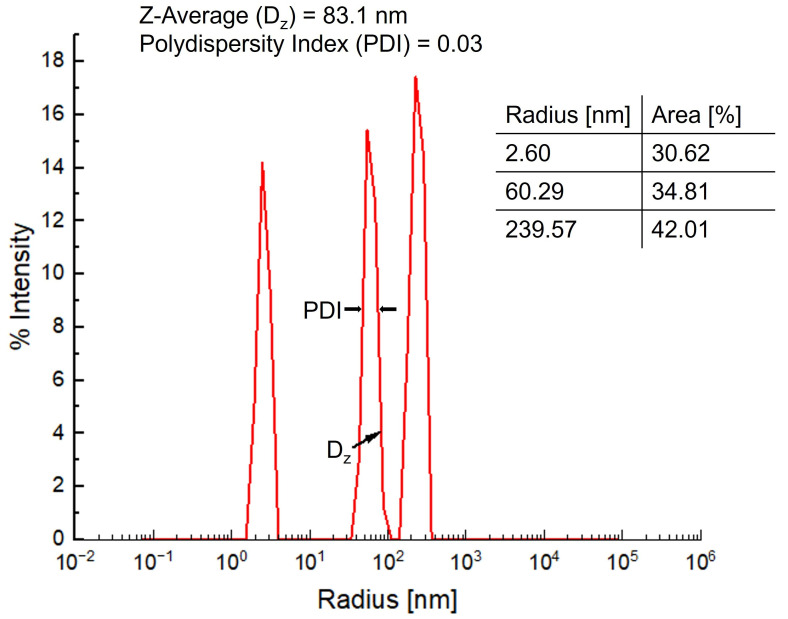
Dynamic light scattering (DLS) intensity size distribution of the fMIP-NP dispersion. The graph shows three distinct populations with average radii of 2.60 nm (30.62%), 60.29 nm (34.81%), and 239.57 nm (42.01%). The overall Z-average radius (Dz) is 83.1 nm, and the polydispersity index (PDI) for each individual peak is 0.03.

The size distribution of the fMIP-NPs was characterized using dynamic light scattering (DLS), as shown in Figure 8. The intensity-weighted distribution profile exhibited three distinct peaks corresponding to radii of 2.60 nm (30.62%), 60.29 nm (34.81%), and 239.57 nm (42.01%). The presence of these multiple size populations indicates a heterogeneous dispersion, potentially consisting of individual nanoparticles, small aggregates, and larger agglomerates. The Z-average radius (Dz), representing the intensity-weighted mean radius of the entire particle population as determined by cumulant analysis, was measured to be 83.1 nm. The low polydispersity index (PDI = 0.03 for each peak) indicates a high degree of monodispersity within each individual population, suggesting a more uniform and homogeneous particle size distribution. The dominant contribution of the larger population (239.57 nm) to scattering intensity, despite likely representing a smaller number fraction, is attributable to the fact that larger particles scatter light more intensely than smaller ones. This intensity-based bias is a known characteristic of DLS measurements and must be considered when interpreting size distributions. Overall, the DLS results confirm that the synthesized fMIP-NPs predominantly consist of nanoscale particles with narrow size distributions, although minor aggregation is evident, likely due to interparticle interactions in the dispersion.

#### 3.5.2. Morphology Analysis by Scanning Electron Microscopy (SEM)

**Figure 9:**
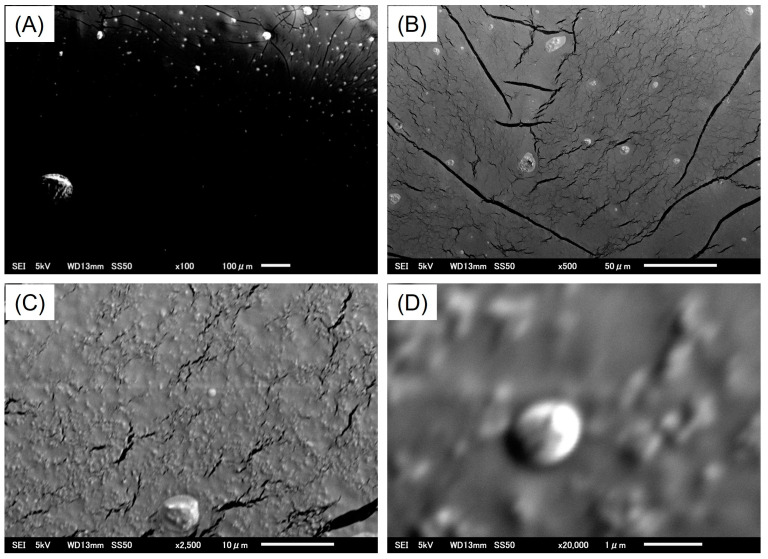
Scanning electron microscopy (SEM) images of dried fMIP-NP dispersed solution, prepared by drying at room temperature prior to imaging. The images show the surface morphology at various magnifications: (**A**) ×100, (**B**) ×500, (**C**) ×2500, and (**D**) ×20,000.

The surface morphology of the synthesized fMIP-NPs was investigated using scanning electron microscopy (SEM), as shown in Figure 9. At low magnification (×100, Figure 9A), the SEM images revealed bright spots with diameters ranging from approximately 1 to 100 µm. These sizes are significantly larger than the nanoscale dimensions determined by DLS, indicating that the observed structures are likely nanoparticle agglomerates formed during the drying process. At intermediate magnifications (×500 and ×2500, Figure 9B,C), the images show pronounced surface cracks alongside the agglomerated nanoparticles. These cracks likely arise from mechanical stresses induced by solvent evaporation and the subsequent shrinkage of the dried particle film. At the highest magnification examined (×20,000, Figure 9D), a spherical agglomerate approximately 1 µm in diameter is observed. Overall, the SEM analysis confirms the spherical morphology of the fMIP-NP agglomerates, likely formed during drying.

### 3.6. Binding Isotherm Analysis of fMIP-NPs with Serotonin

**Figure 10:**
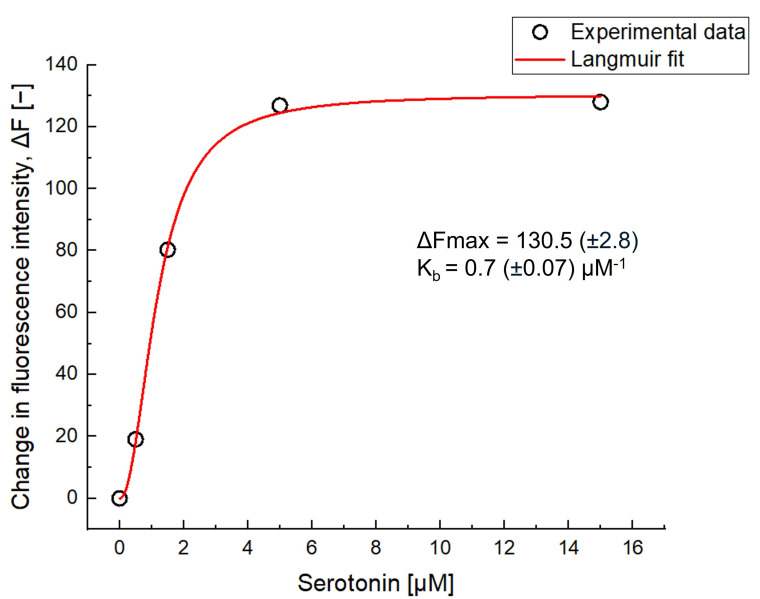
Binding isotherm of serotonin with fMIP-NPs fitted using the Langmuir model.

The binding isotherm of serotonin to the fluorescent molecularly imprinted polymer nanoparticles (fMIP-NPs) was constructed based on the assumption that the change in fluorescence intensity (Δ*F*) is directly proportional to the amount of serotonin bound. As shown in Figure 10, a Langmuir binding model was applied to the experimental data to evaluate the binding affinity and saturation behavior of the fMIP-NPs.

From the Langmuir fit, the maximum fluorescence change (Δ*F*max) was estimated to be 130.5, representing the saturation level of the available binding sites on the nanoparticles. The binding constant (*K*_b_) was calculated to be 0.7 μM^−1^, corresponding to a dissociation constant (*K*_d_) of approximately 1.43 μM (*K*_d_ = 1/*K*_b_). These values indicate that the fMIP-NPs exhibit moderate to high affinity for serotonin under the experimental conditions. The shape of the isotherm closely follows typical Langmuir behavior, suggesting monolayer adsorption onto a homogeneous surface with a finite number of binding sites. The dissociation constant for the subsequent batch was considered unreliable, as it was derived from a response scale with lower sensitivity and precision; therefore, it is not discussed further. The K_d_ value of the fMIP-NP is much higher than that of antibodies (which are typically in the picomolar to nanomolar range [44]) or aptamers (in the nanomolar range [45]) but is similar to that of usual MIPs [46,47].

## 4. Conclusions

In this study, we demonstrated that poly(methacrolein-*co*-methacrylamide) crosslinking between the template and solid surface is effective for synthesizing highly selective fMIP-NPs for serotonin detection. By optimizing the aldehyde content in poly(methacrolein-*co*-methacrylamide), we were able to enhance both the selectivity and sensitivity of the fMIP-NPs, resulting in a 37% increase in fluorescence intensity upon serotonin binding. These findings suggest that the developed fMIP-NPs can serve as promising probes for neurotransmitter imaging applications. While our current results are limited to preliminary validation, future research will focus on evaluating the applicability of fMIP-NPs as imaging probes in living animal neurons, for example, in model organisms such as *Aplysia*. Nevertheless, systematic in vitro validation using isolated neurons will be essential to confirm the feasibility and effectiveness of this approach.

## 5. Patents

We have a patent for Molecularly Imprinted Nanoparticles with Fluorescent Functional Groups (JP2018132527A).

## Figures and Tables

**Table 1:** Synthesis conditions for poly(methacrolein-*co*-methacrylamide).

	Molar Ratio of Methacrolein to Methacrylamide	Molar Ratio of Initiator to Total Comonomer	Total Comonomer Concentration [M]
Condition 1	1:3	1:100	1.23
Condition 2	1:1	1:100	4.30
Condition 3	5:1	1:150	4.59

**Table 2:** Comparison of the moles of aldehyde groups and the estimated molar ratio of aldehyde to amine groups in the copolymer synthesized under Conditions 1–3, based on titration experiments.

	Molar Ratio of Methacrolein to Methacrylamide in Pre-Polymer Solution	Moles of -(CH_2_C(CH_3_)-CHO)- in 0.5 g of Synthesized Copolymer [µmol]	Molar Ratio of -(CH_2_C(CH_3_)-CHO)- to -(CH_2_C(CH_3_)-NH_2_)- in the Synthesized Copolymer
Condition 1	1:3	4.5	7.7 × 10^−3^
Condition 2	1:1	9.3	1.5 × 10^−2^
Condition 3	5:1	38.8	7.1 × 10^−2^

## Data Availability

Data is contained within the article or Supplementary Material. The data supporting the findings of this study are available from the corresponding author upon reasonable request, subject to a written agreement.

## Supplementary Materials

The following supporting information can be downloaded at: https://www.mdpi.com/article/10.3390/nano15130977/s1, Figure S1. (A) Fluorescence emission spectra of fMIP-NPs synthesized in a subsequent batch prepared 8 months later, using serotonin immobilized on glass beads via a poly(methacrolein-co-methacrylamide) anchor under Condition 3, measured at varying serotonin (5-HT) concentrations. (B) Effect of serotonin (5-HT, circles) and its analogue L-tryptophan (L-Trp, triangles) on the fluorescence intensity of the corresponding fMIP-NPs under the same condition.
