# Supplementary material for: A Poly(methacrolein-co-methacrylamide)-Based Template Anchoring Strategy for the Synthesis of Fluorescent Molecularly Imprinted Polymer Nanoparticles for Highly Selective Serotonin Sensing
Source: Nanomaterials (Basel). 2025 Jun 24;15(13):977. doi: 10.3390/nano15130977 (PMC12251135; doi:10.3390/nano15130977)
Supplement: Supplementary file 1 [file nanomaterials-15-00977-s001.zip › nanomaterials-3651629-supplementary.pdf]

Supplementary Figure :

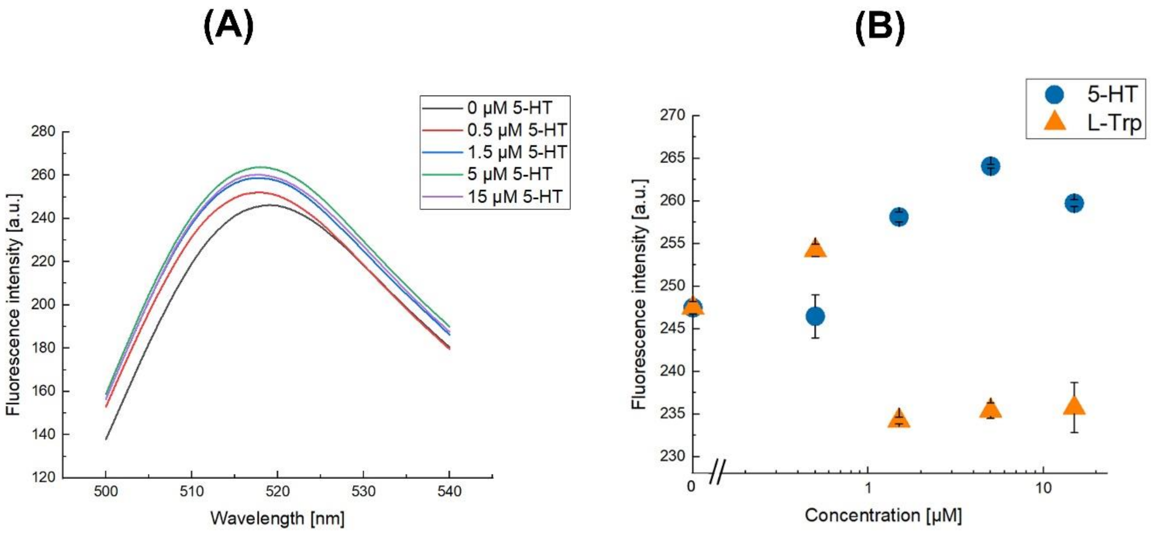

Figure S1. (A) Fluorescence emission spectra of fMIP-NPs synthesized in a subsequent batch prepared 8 months later, using serotonin immobilized on glass beads via a poly(methacrolein-co-methacrylamide) anchor under Condition 3, measured at varying serotonin (5-HT) concentrations. (B) Effect of serotonin (5-HT, circles) and its analogue L-tryptophan (L-Trp, triangles) on the fluorescence intensity of the corresponding fMIP-NPs under the same condition.
